# Conduction System Pacing Versus Biventricular Cardiac Resynchronization Pacing: Meta-Analysis on Outcomes in Patients with Non-Left Bundle Branch Block

**DOI:** 10.3390/medicina61071240

**Published:** 2025-07-09

**Authors:** Xuanming Pung, Joe J. L. Chua, Khi Yung Fong, Yi Yi Chua, Germaine J. M. Loo, Jonathan W. S. Ong, Julian C. K. Tay, Hooi Khee Teo, Yue Wang, Colin Yeo, Eric T. S. Lim, Kah Leng Ho, Daniel T. T. Chong, Chi Keong Ching, Vern Hsen Tan

**Affiliations:** 1 Department of Cardiology, National Heart Centre Singapore, Singapore 169609, Singapore; pung.xuanming@singhealth.com.sg (X.P.);; 2 Department of General Medicine, Changi General Hospital, Singapore 529889, Singapore; 3 Department of Cardiology, Changi General Hospital, Singapore 529889, Singapore

**Keywords:** conduction system pacing, cardiac physiologic pacing, biventricular pacing, cardiac resynchronization therapy, right bundle branch block, intraventricular conduction delay

## Abstract

*Background and Objectives*: The role of biventricular pacing (BVP) is less well-established in patients with heart failure with reduced ejection fraction (HFrEF) without left bundle branch block (LBBB). Conduction system pacing (CSP) has gained significant traction and may provide a safe and more physiological alternative to BVP in these patients. A few small studies studying this question have reported conflicting results. This meta-analysis aims to compare procedural and clinical outcomes between CSP and BVP in this group. *Materials and Methods*: An online literature search was systematically conducted to retrieve studies comparing CSP and BVP in HFrEF patients with non-LBBB. Four studies with 461 patients were included. *Results*: Implant-derived paced QRS duration was significantly shorter (mean difference [MD] −19.7 ms, 95% confidence interval [CI] −36.2 to −3.3, *p* = 0.0355) with CSP. Echocardiographic response with significantly greater improvement in left ventricular ejection fraction (MD 5.6%, 95% CI 3.1 to 8.0, *p* = 0.0106) was also observed with CSP. There were no statistically significant differences in clinical outcomes such as all-cause mortality (relative risk [RR] 0.53, 95% CI 0.18 to 1.60, *p* = 0.133) and heart failure hospitalization (RR 0.54, 95% CI 0.19 to 1.56, *p* = 0.129). *Conclusions*: This meta-analysis suggests that CSP may have better electrical synchrony and echocardiographic response compared to BVP in HFrEF patients with non-LBBB. Further randomized studies with longer follow-up may be required to elucidate potential benefits in clinical outcomes.

## 1. Introduction

Cardiac physiologic pacing refers to any form of cardiac pacing intended to restore or preserve synchrony of ventricular contraction. In patients with heart failure with reduced ejection fraction (HFrEF), this can be achieved by either engaging the intrinsic conduction system via conduction system pacing (CSP) or biventricular pacing (BVP) with a coronary sinus branch or left epicardial or endocardial lead [1].

Extensive evidence and guidelines support the use of BVP as cardiac resynchronization therapy (CRT) in HFrEF patients [1,2]. However, the electromechanical and clinical benefits attained from BVP appear largely limited to patients with left bundle branch block (LBBB) morphology [3]. In comparison, CSP—encompassing His-bundle pacing (HBP) and left bundle branch pacing (LBBP)—has been shown in a recent meta-analysis of randomized controlled trial to be superior to BVP with respect to shortening of QRS duration, improvement in left ventricular ejection fraction, and improvement in New York Heart Association (NYHA) class [4]. However, whether this extends to the non-LBBB group is unclear.

One clear challenge of CRT pertains to pre-existing non-LBBB electrocardiogram patterns [5]. To address this question, we conducted a meta-analysis of existing studies to evaluate electrocardiographic, echocardiographic, and clinical outcomes of CSP versus BVP in HFrEF patients with non-LBBB.

## 2. Materials and Methods

### 2.1. Literature Search

This systematic review and meta-analysis were conducted in accordance with the Preferred Reporting Items for Systematic Reviews and Meta-Analyses (PRISMA) guidelines and registered with the International Prospective Register of Systematic Reviews (PROSPERO; identifier: CRD420251051545) [6].

An electronic literature search from inception until 30 April 2024 was conducted by two independent investigators (K.Y.F. and V.H.T.) on PubMed, Embase, and Scopus for relevant articles using the concepts of CSP, HBP, LBBP, non-LBBB, and HFrEF. The full search strategy is shown in Table S1. The bibliographies of included studies were screened, and a search on Google Scholar using the first and last authors of each included study was conducted to ensure inclusion of all relevant studies. Retrieved abstracts and full texts were reviewed by two independent investigators (K.Y.F. and V.H.T.), with conflicts being resolved via group consensus among all authors.

Prospective or retrospective studies reporting comparisons of the procedural and clinical outcomes between HFrEF patients with non-LBBB who underwent CSP versus BVP were included. Similar studies that studied HFrEF patients regardless of QRS morphology but reported group data for the non-LBBB cohort were also included. Case reports, case series, reviews, and conference abstracts were excluded. Studies that did not report group data for the non-LBBB cohort were also excluded, e.g., [7].

A standardized data collection template with predefined data fields, including study characteristics, patient demographics, and outcomes, was used for data extraction by two independent investigators. Studies were assessed for risk of bias using the Newcastle–Ottawa scale [8].

### 2.2. Meta-Analysis

The primary outcomes analyzed in this meta-analysis were all-cause mortality, heart failure hospitalization, QRS duration post-implant, and change in left ventricular ejection fraction (LVEF). For continuous outcomes, means and standard deviations were pooled in random-effects meta-analysis to determine mean differences and 95% confidence intervals (CIs). For binary outcomes, numbers of patients per arm and event counts were pooled in random-effects meta-analysis to determine risk ratios and 95% CIs. Missing means and standard deviations were derived from medians and interquartile ranges in accordance with established methods reported by Hozo et al. and Wan et al. [9,10].

Study heterogeneity was stratified as low, moderate, or considerable for *I*^2^ values < 40%, 40–75%, and >75%, respectively [11]. Funnel plot symmetries were visually assessed for publication bias. Certainty of evidence was evaluated using the GRADE approach [12]. All statistical analyses were performed with R version 4.3.0 (R Core Team, Vienna, Austria). *p* < 0.05 was considered to indicate statistical significance.

## 3. Results

### 3.1. Study Selection

**Figure 1:**
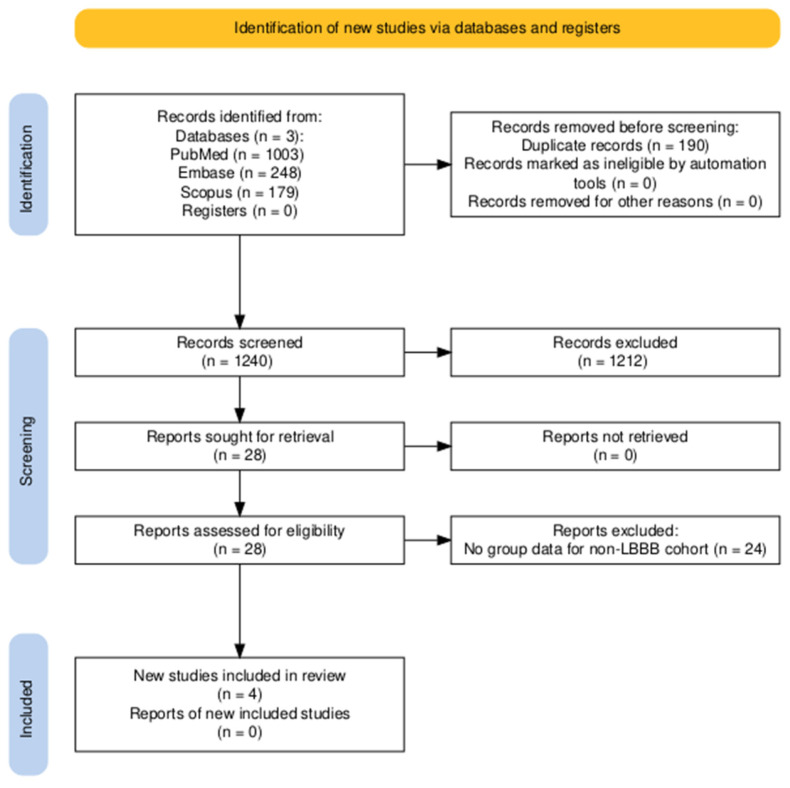
PRISMA flow diagram for the included studies.

The initial search contained 1430 studies. After the removal of 190 duplicates, 1240 studies underwent title and abstract screening. Twenty-eight studies were then selected for full-text review, with a final four studies consisting of 461 patients selected for analysis [13,14,15,16]. The PRISMA flow diagram is shown in Figure 1 [17].

### 3.2. Study Characteristics

The included studies were published between 2022 and 2023, due to the recent increased widespread adoption of CSP and the limited number of eligible studies meeting our criteria. All studies were non-randomized; three were retrospective [13,14,15] and one prospective [16]. Vijayaraman et al. only reported data for the overall cohort and the LBBB group; however, the baseline characteristics, event rates, and hazard ratios for the non-LBBB group were computable from the provided data and were included in the final meta-analysis [13].

A total of 461 patients were studied, with 255 undergoing CSP and 206 undergoing BVP. Baseline characteristics of the included studies are shown in Table 1. The average age of the cohort was 75, with 75% being male. One study only included patients with AV block [14], another only intraventricular conduction delay (IVCD) [16], and the remaining two a mix of patients with right bundle branch block (RBBB), IVCD, AV block, and normal QRS [13,15]. With regards to the method of CSP, two studies used a mix of HBP and LBBP [14,15], one purely used left bundle branch optimized CRT (LOT-CRT) [16], and the last used a mix of all three [13]. The average follow-up duration was variable, ranging from 365 to 810 days. The risk of bias was generally low (Table S2).

### 3.3. Statistical Analysis

**Figure 2:**
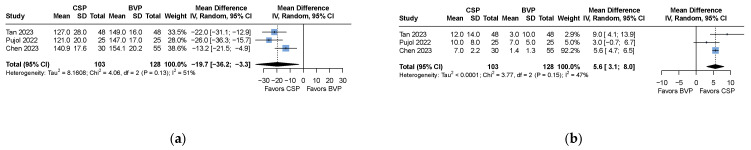
Meta-analysis of (**a**) QRS duration and (**b**) LVEF improvement [14,15,16].

Post-implant QRS duration and LVEF improvement were described in three studies [14,15,16]. The relevant forest plots are shown in Figure 2. Implant-derived paced QRS duration was significantly shorter with CSP (mean difference [MD] −19.7 ms, 95% confidence interval [CI] −36.2 to −3.3, *p* = 0.0355, *I*^2^ = 51%). An echocardiographic response with significantly greater improvement in LVEF was also observed in CSP (MD 5.6%, 95% CI 3.1 to 8.0, *p* = 0.0106, *I*^2^ = 47%).

**Figure 3:**
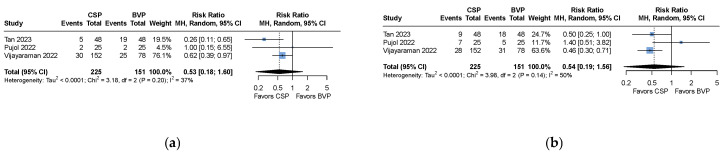
Meta-analysis of (**a**) all-cause mortality and (**b**) heart failure hospitalization [13,14,15].

Clinical outcomes of interest (all-cause mortality and heart failure hospitalization) were described in three studies [13,14,15]. There were no statistically significant differences in both outcomes (all-cause mortality: relative risk [RR] 0.53, 95% CI 0.18 to 1.60, *p* = 0.133, *I*^2^ = 37%; heart failure hospitalization: RR 0.54, 95% CI 0.19 to 1.56, *p* = 0.129, *I^2^* = 50%), as seen in Figure 3.

Funnel plots for all outcomes were not visually suggestive of publication bias (Figures S1–S4). The GRADE ratings are shown in Table 2.

## 4. Discussion

### 4.1. Difference in Outcomes Between CSP and BVP in Non-LBBB

This meta-analysis demonstrates that in HFrEF patients with non-LBBB, CSP significantly shortened QRS duration and improved LVEF but had no difference in clinical outcomes (heart failure hospitalization or all-cause mortality).

Currently, LBBB represents an important electrophysiological substrate for CRT response. Multiple criteria for LBBB have been published to facilitate CRT decisions, with major differences between them [2,18,19,20]. In a study by van Stipdonk and colleagues, LBBB classification was shown to be highly dependent on the criterion used, and only 13.8% of patients satisfied all LBBB criteria [21]. Despite the significant differences in classified LBBB morphology among the studied criteria, the investigators concluded that LBBB was still associated with significant differences in survival free of primary endpoints, regardless of the criterion used. On the other hand, outcomes in non-LBBB patients are less impressive, with only a class 2A recommendation in patients with NYHA class III or greater symptoms [1].

The recent CONSYST-CRT trial demonstrated the clinical and echocardiographic non-inferiority of CSP compared to BVP in patients with an indication for CRT, suggesting that CSP could be an alternative to BVP in all-comers requiring CRT [22]. However, supporting studies specifically examining the non-LBBB group remain scarce, and to date, there are no randomized controlled trials studying this population. As such, the use of CSP in such patients is only supported by a class 2B recommendation (may be reasonable) in contemporary guidelines, and only when effective CRT cannot be achieved with BVP [1].

In our meta-analysis, we analyzed a total of four observational studies (one specifically for non-LBBB patients, and three with outcome data reported for non-LBBB patients) and found that although CSP was associated with shorter QRS durations and greater improvements in LVEF, this did not translate into better clinical outcomes (reduced all-cause mortality or heart failure hospitalization), potentially due to the short follow up durations in the included studies. Of note, a recent meta-analysis also failed to demonstrate a difference in clinical outcomes between CSP and BVP in all-comers; however, follow-up durations in the included studies were also relatively short (ranging from 6 to 12 months) [4].

Interestingly, although the improvement in clinical outcomes in the pooled meta-analysis did not reach significance, there was a signal towards improvement, mainly driven by two out of three of the included studies [13,15]. The third study reported equivocal improvements in clinical outcomes with CSP; however, this may have been influenced by the shorter follow-up (6 months) and the smaller sample size (*n* = 50) [14]. Additionally, this third study only included patients with a permanent pacing indication due to atrioventricular (AV) block, whereas the other two included patients with prolonged QRS durations due to RBBB and IVCD, as well as patients who required pacing due to AV block. At present, the choice of pacing modality in patients with AV block, a narrow QRS complex, and HFrEF is still controversial [23]. Further work is needed to explore the effect of CSP and BVP on the different subgroups of non-LBBB.

Meta-analyses have previously demonstrated that BVP does not reduce death and heart failure hospitalizations in non-LBBB patients, and CSP may supplant BVP as the preferred pacing or CRT method in such patients if robust clinical benefits are demonstrated [3]. In addition, novel techniques like His-optimized and left bundle branch-optimized CRT have shown promise, with greater electrocardiographic, echocardiographic, and clinical response compared to traditional His bundle pacing [24,25].

### 4.2. Choosing Between CSP and BVP in Non-LBBB

**General procedural outcomes.** A study by Sharma et al. studied HBP in patients with HFrEF, RBBB, and QRS duration ≥120 ms. The thresholds for His capture and bundle branch block correction were 1.1 ± 0.6 and 1.4 ± 0.7 V at 1.0 ms, respectively. HBP resulted in a shortening of the QRS duration from 158 ± 24 to 127 ± 17 ms (*p* = 0.0001), increase in LVEF from 31 ± 10% to 39 ± 13% (*p* = 0.004), and improvement in NYHA class from 2.8 ± 0.6 to 2 ± 0.7 (*p* = 0.0001) after a mean follow-up of 15 ± 23 months [26]. Another observational study by Vijayaraman et al. studying LBBP in a similar cohort with indications for CRT or pacing resulted in thresholds of 0.8 ± 0.3 V at 0.5 ms at implantation, which remained stable after a mean follow-up period of 13 ± 8 months. With LBBP, there was a decrease in QRS duration from 156 ± 20 ms at baseline to 150 ± 24 ms (*p* = 0.01), increase in LVEF from 35 ± 9% to 43 ± 12% (*p* < 0.01), and improvement in NYHA class from 2.5 ± 0.8 at baseline to 1.7 ± 0.8 (*p* < 0.01). However, when compared to LBBB, a non-LBBB QRS morphology itself has been shown to triple the likelihood of failed LBBP implantation [27]. Female sex and reduction of QRS duration with LBBP were associated with echocardiographic response and super-response [28].

**QRS duration and morphology.** The clinical and echocardiographic responses and benefits attained with CSP may differ for specific types of non-LBBB, with IVCD representing a particularly challenging substrate for CSP. Upadhyay and colleagues first demonstrated that HBP did not correct QRS duration in patients with intact Purkinje activation/IVCD [29]. Most recently, out of the 21 patients with IVCD included in the CONSYST-CRT trial, nine of the eleven patients randomized to CSP required crossover, suggesting suboptimal procedural or clinical outcomes in this group [22]. These difficulties, however, do not appear to extend to other non-LBBB morphologies; a study by Vijayaraman et al. showed that the rate of unsuccessful LBBP attempts was high in the IVCD group (*p* = 0.02) but not in the RBBB group (*p* = 0.81) [30]. Ultimately, QRS duration has been shown to impact outcomes regardless of non-LBBB morphology. A meta-analysis of five randomized clinical trials highlighted the importance of having a prolonged QRS duration at baseline for CRT to be beneficial in non-LBBB, with its multivariate model highlighting that the sole predictor of CRT effect on outcomes was a QRS duration > 140 ms. Further analysis also demonstrated increasing CRT benefits with increasing QRS duration on all-cause mortality and the composite of first hospitalization for heart failure or death, independent of QRS morphology [31].

**AV block.** The presence of first-degree AV block has been shown to be an important factor in non-LBBB for patients to benefit from CRT in a sub-study of the Multicenter Automatic Defibrillator Implantation Trial-Cardiac Resynchronization Therapy (MADIT-CRT) trial. This study showed that patients with a non-LBBB pattern and prolonged PR interval ≥230 ms demonstrated significant improvement in clinical endpoints with CRT, including reductions in the risk of heart failure and all-cause mortality. Conversely, PR interval duration was not an important consideration in HBP, as positive clinical and echocardiographic benefits were similarly observed in patients with normal PR interval [32]. Notably, HBP has been shown in a recent clinical trial to result in improvements in symptoms and quality of life when compared to no pacing in patients with HFrEF, PR interval ≥200 ms, and either QRS ≤ 140 ms and right bundle branch block [33]. However, HBP may not be suitable in patients with infra-nodal AV block or scheduled AV node ablation [34].

**Myocardial scarring.** Anatomical factors should also be considered when deciding between CSP and BVP in patients without LBBB. In a study by Ponnusamy et al., findings of transmural scar in the LBBP zone via late gadolinium enhancement on cardiac magnetic resonance imaging predicted procedural failure with 100% sensitivity and 100% specificity [35]. Elliott MK et al. also demonstrated superior electrical resynchronization and greater acute hemodynamic response during biventricular endocardial pacing and LBBP compared to biventricular epicardial pacing. Electrical resynchronization was similar between biventricular endocardial pacing and LBBP; however, the septal scar seemed to attenuate the response to LBBP, possibly due to increased difficulty in penetrating the septum [36].

**Valve pathology or planned intervention.** The presence of tricuspid regurgitation should prompt further deliberation regarding the modality of pacing. Basal septal LBBP lead placement close to the tricuspid annulus has previously been shown to worsen tricuspid regurgitation, with proposed cutoffs of 16 or 19 mm from the annulus [37,38]. Surgical or percutaneous tricuspid valve interventions, if required, may also lead to inadvertent jailing of the LBBP lead and/or lead damage. In cases where there is tricuspid valve dysfunction, previous transcatheter repair, or a prosthesis in situ, HBP or BVP may be preferred over LBBP in providing electrical resynchronization. On the other hand, HBP may be less preferred in previous or scheduled transcatheter aortic valve implantation or aortic valve surgery due to the risk of compromised HBP lead function following these interventions [34]. Notably, a recent first-in-human study of a leadless LBBP system was recently reported and may represent a solution to these anatomical issues [39].

**Summary.** From the studies described above, it appears that CSP may be preferred over BVP in non-LBBB if the QRS duration is ≤140 ms or the PR interval is <230 ms, and in the absence of a significant IVCD pattern or specific anatomical contraindications. Future randomized studies focused on non-LBBB patients could elucidate potential clinical benefits of CSP compared to BVP in this patient group. Further data describing the long-term stability of CSP and capabilities in the recruitment of local conduction system tissue are also needed, particularly in the presence of His-Purkinje conduction disease.

### 4.3. Limitations

Our meta-analysis included only observational studies, as there were no randomized studies that matched our inclusion criteria. As a result, the GRADE ratings for certainty of evidence ranged from low to very low, highlighting the urgent need for future randomized studies. Study designs, sample sizes, and duration of follow-up were also heterogeneous within the included studies, which may affect the generalizability of our results. The types of non-LBBB patterns included were different between studies; future studies focusing on specific types of non-LBBB (e.g., RBBB, IVCD with and without concomitant AV block indication for pacing) would greatly clarify the distinction between these groups.

## 5. Conclusions

This meta-analysis suggests that CSP may have better electrical synchrony and echocardiographic response compared to BVP in HFrEF patients with non-LBBB. However, there were no significant differences in clinical outcomes such as all-cause mortality and heart failure hospitalization. Future randomized studies are needed to further elucidate the potential benefits of CSP in this group.

## Figures and Tables

**Table 1:** Baseline characteristics of included studies.

Reference	Study Type	Non-LBBB Type	CSP Type	Arm	*n*	Age, y *	Male, %	HTN, %	DM, %	IHD, %	AF, %	CKD, %	NYHA III–IV, %	Follow Up, Days
Vijayaraman et al., 2022 [13]	Retrospective	RBBB (19%), IVCD (19%), AVB (40%), normal QRS (21%)	HBP, LBBP, LOT-CRT	CSP	152	73 ^†^	70	69	37	54	66	NR	NR	810
				BVP	78	74 ^†^	73	77	51	63	60	NR	NR	
Pujol-Lopez et al., 2022 [14]	Retrospective	AVB	HPB (72%), LBBP (28%)	CSP	25	72 (9)	68	NR	NR	NR	16	NR	64	365
				BVP	25	69 (8)	76	NR	NR	NR	16	NR	72	
Tan et al., 2023 [15]	Retrospective	RBBB (51%), IVCD (13%), AVB (36%)	HBP, LBBP	CSP	48	70 (10)	77	73	50	63	52	48	NR	578
				BVP	48	70 (12)	80	73	60	73	46	52	NR	728
Chen et al., 2023 [16]	Prospective	IVCD	LOT-CRT	CSP	30	64 (13)	77	30	20	NR	37	NR	63	730
				BVP	55	64 (11)	87	27	35	NR	26	NR	71	

* Reported as mean (SD). ^†^ The standard error was not computable from the data provided. AF: atrial fibrillation; AVB: atrioventricular block; CSP: conduction system pacing; CKD: chronic kidney disease; DM: diabetes mellitus; HBP: His bundle pacing; HTN: hypertension; IHD: ischemic heart disease; IVCD: intraventricular conduction delay; LBBP: left bundle branch pacing; LOT-CRT: left bundle branch optimized cardiac resynchronization therapy; NR: not reported; NYHA: New York Heart Association; RBBB: right bundle branch block.

**Table 2:** The GRADE table summary. CI: confidence interval; LVEF: left ventricular ejection fraction; MD: mean difference; RR: relative risk.

Outcome	Relative Effect (95% CI)	Number of Participants (Studies)	Heterogeneity, %	Certainty of Evidence (GRADE)
QRS duration	MD −19.7 (−36.2 to −3.3)	231 (3 studies)	51	⊕⊖⊖⊖ Very low *
LVEF improvement	MD 5.6 (3.1 to 8.0)	231 (3 studies)	47	⊕⊖⊖⊖ Very low *
All-cause mortality	RR 0.53 (CI 0.18 to 1.60)	376 (3 studies)	37	⊕⊕⊖⊖ Low
Heart failure hospitalization	RR 0.54 (0.19 to 1.56)	376 (3 studies)	50	⊕⊖⊖⊖ Very low *

* Downgraded one level due to moderate heterogeneity.

## Data Availability

No new data were created or analyzed in this study. Data sharing is not applicable to this article.

## Supplementary Materials

The following supporting information can be downloaded at: https://www.mdpi.com/article/10.3390/medicina61071240/s1, Figure S1: Funnel plot for paced QRS duration; Figure S2: Funnel plot for improvement in LVEF; Figure S3: Funnel plot for all-cause mortality; Figure S4: Funnel plot for heart failure hospitalization; Table S1: Full search phrases used for the respective databases; Table S2: Risk of bias in included studies.

## Abbreviations

The following abbreviations are used in this manuscript:

AV	Atrioventricular
BVP	Biventricular pacing
CI	Confidence interval
CRT	Cardiac resynchronization therapy
CSP	Conduction system pacing
HBP	His bundle pacing
HFrEF	Heart failure with reduced ejection fraction
HOT-CRT	His bundle optimized cardiac resynchronization therapy
IVCD	Intraventricular conduction delay
LBBB	Left bundle branch block
LBBP	Left bundle branch pacing
LOT-CRT	Left bundle branch optimized cardiac resynchronization therapy
LVEF	Left ventricular ejection fraction
MD	Mean difference
NYHA	New York Heart Association
RR	Relative risk
RBBB	Right bundle branch block
