# Supplementary material for: Conduction System Pacing Versus Biventricular Cardiac Resynchronization Pacing: Meta-Analysis on Outcomes in Patients with Non-Left Bundle Branch Block
Source: Medicina (Kaunas). 2025 Jul 9;61(7):1240. doi: 10.3390/medicina61071240 (PMC12299654; doi:10.3390/medicina61071240)

Supplementary Figure S1. Funnel plot for QRS duration

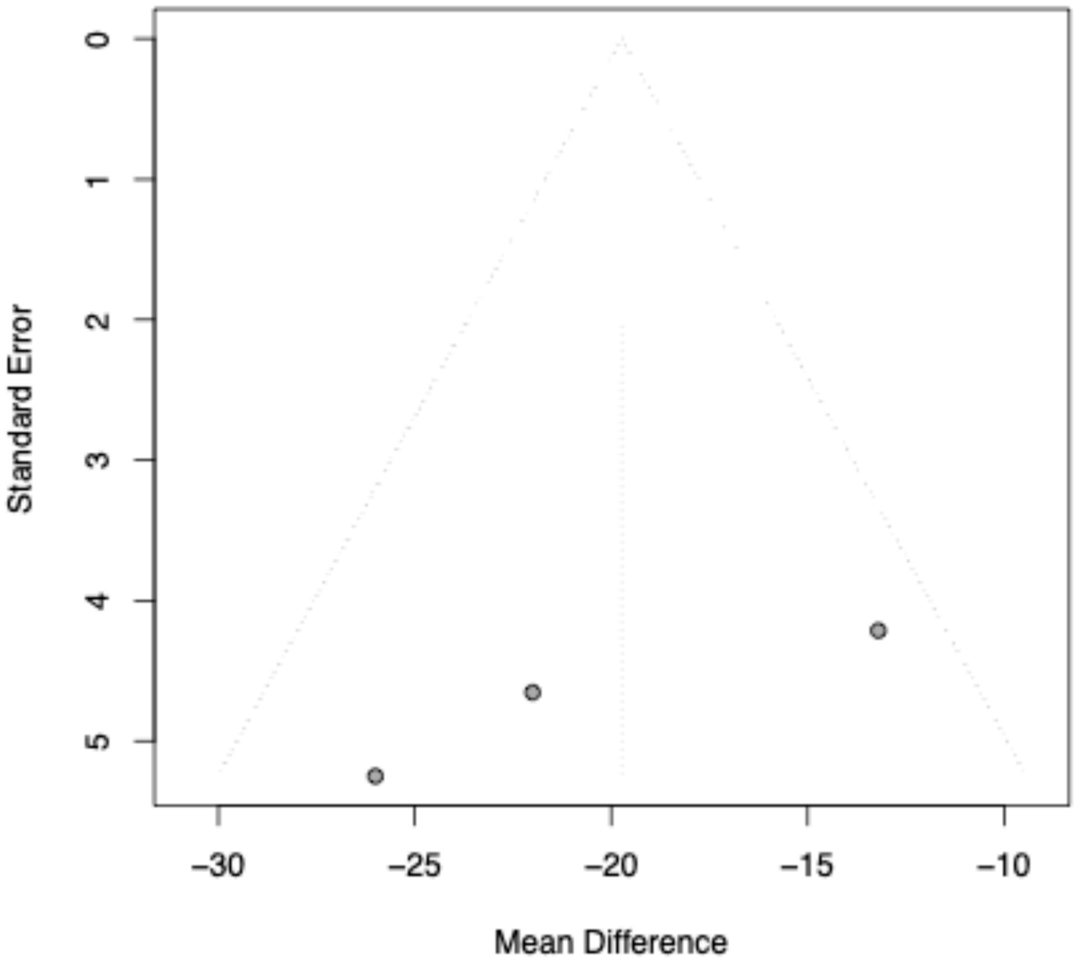

Supplementary Figure S2. Funnel plot for LVEF improvement

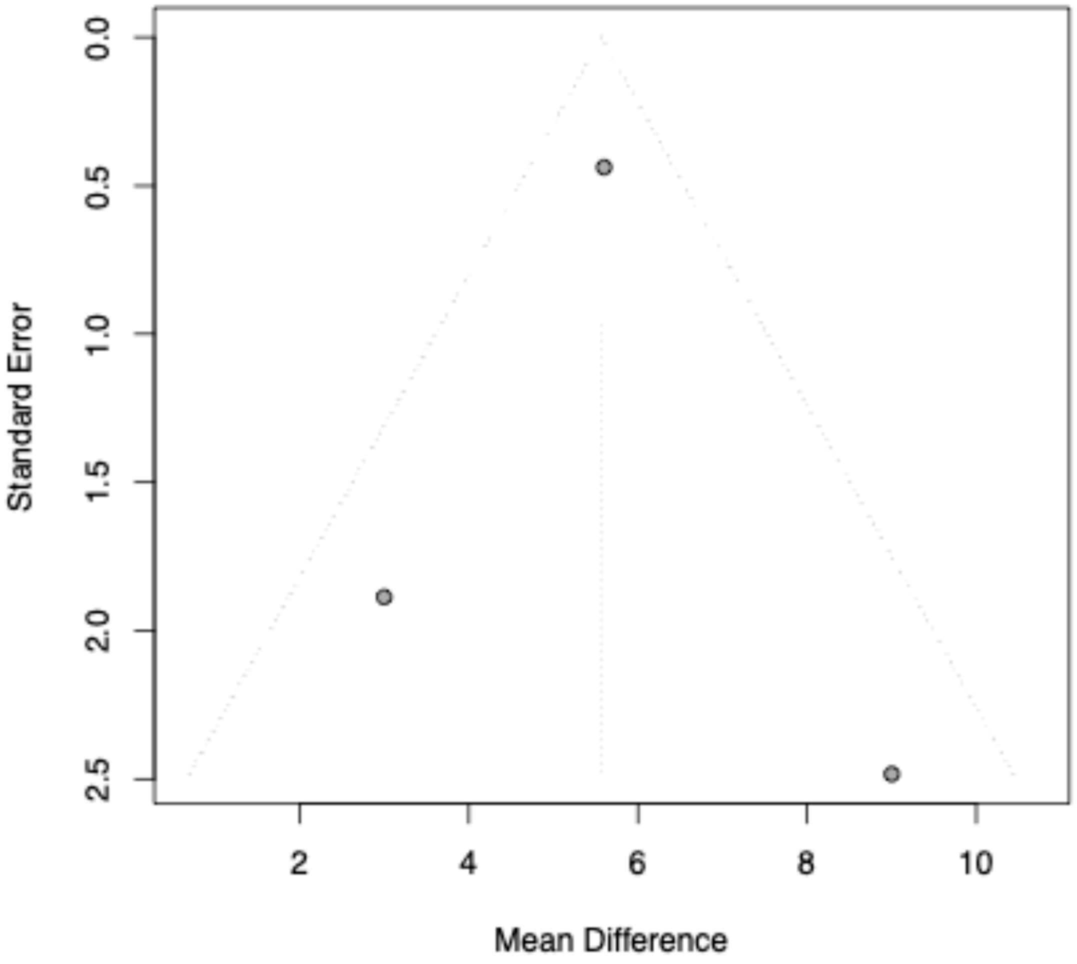

Supplementary Figure S3. Funnel plot for all-cause mortality

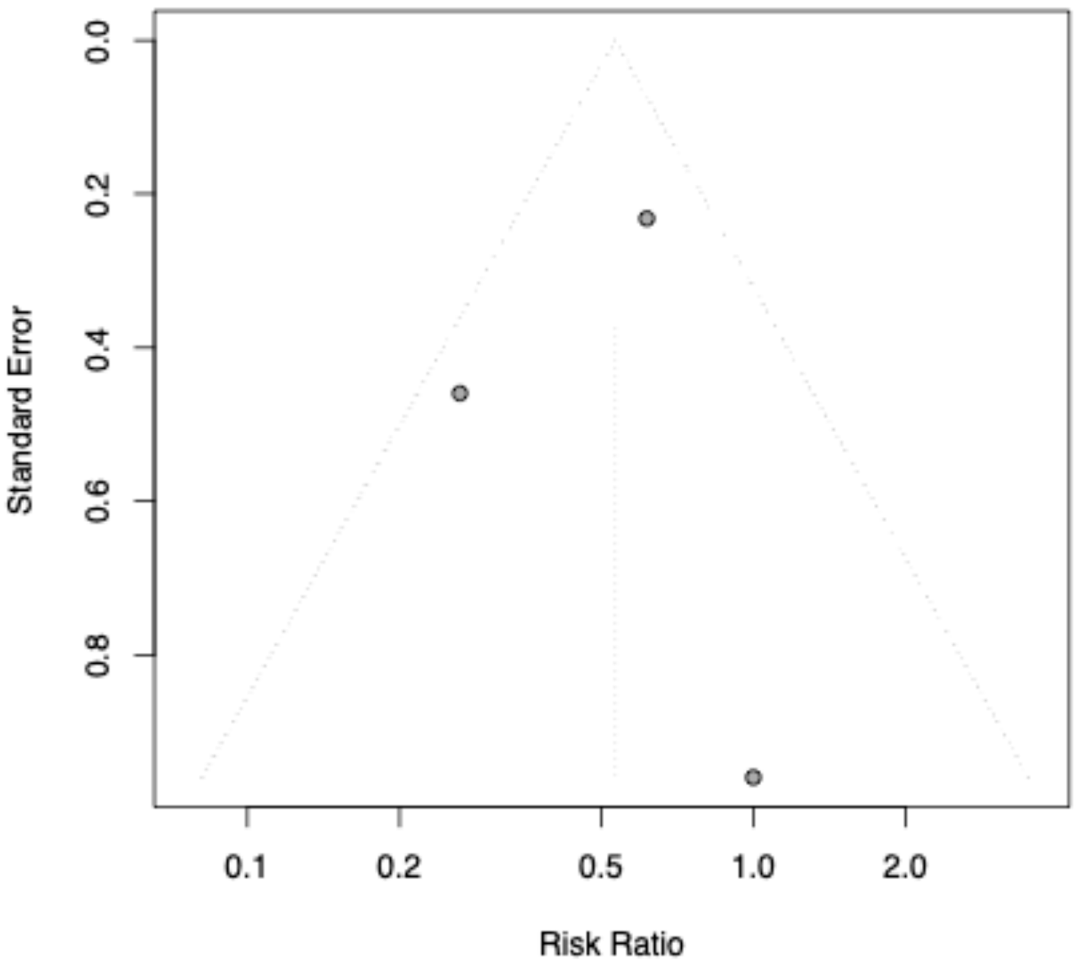

Supplementary Figure S4. Funnel plot for heart failure hospitalization

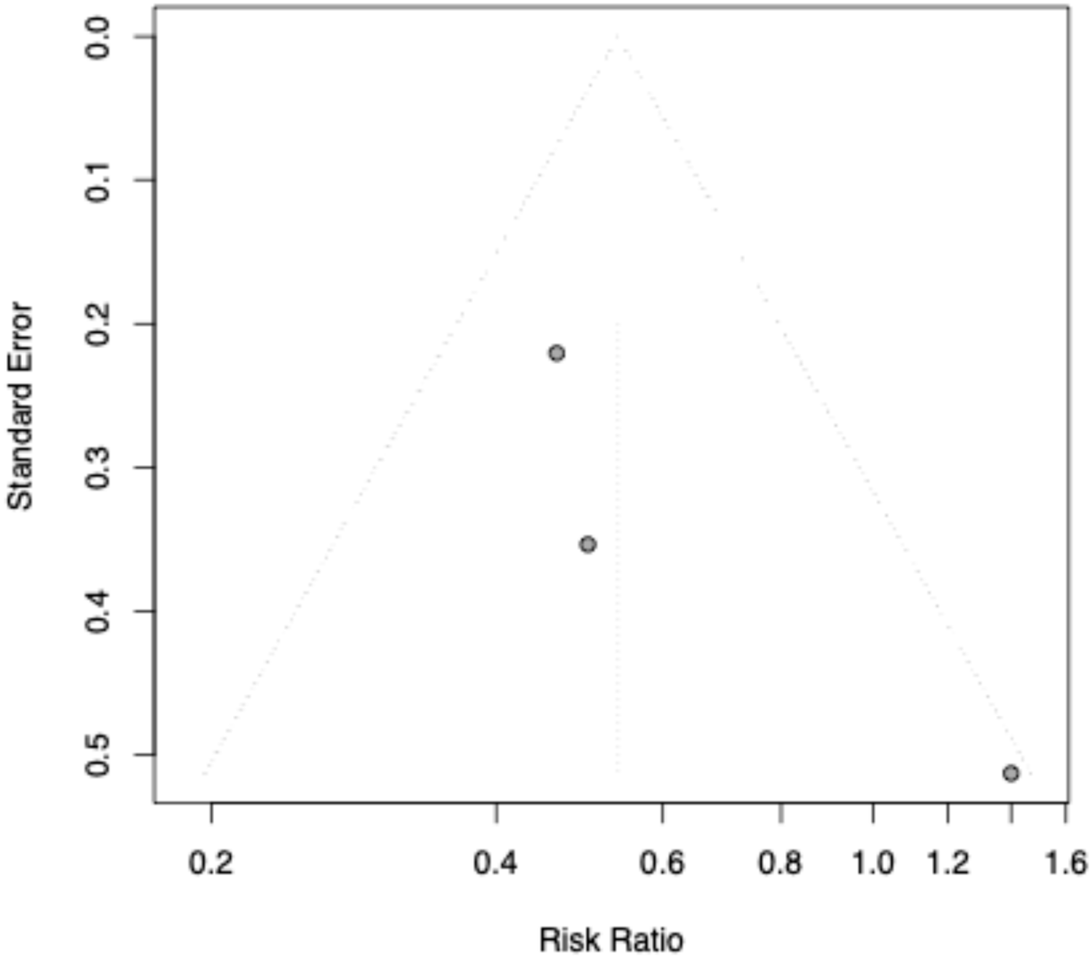

Supplement: Supplementary file 1 [file medicina-61-01240-s001.zip › figures.pdf]
