# Supplementary material for: Conduction System Pacing Versus Biventricular Cardiac Resynchronization Pacing: Meta-Analysis on Outcomes in Patients with Non-Left Bundle Branch Block
Source: Medicina (Kaunas). 2025 Jul 9;61(7):1240. doi: 10.3390/medicina61071240 (PMC12299654; doi:10.3390/medicina61071240)
Supplement: Supplementary file 1 [file medicina-61-01240-s001.zip › tables.pdf]

Supplementary Table S1. Full search phrases used for the respective databases.

**PubMed**	1003 articles
(conduction system pacing OR biventricular pacing OR left bundle branch pacing OR his bundle pacing) AND (RBBB or right bundle branch block OR IVCD OR intraventricular conduction delay OR “non-LBBB”) AND (“Cardiac Resynchronization Therapy”[MeSH] OR “Cardiac Resynchronization Therapy Devices”[MeSH]) NOT systematic[sb] NOT (Review[pt] OR Editorial[pt] OR Letter[pt] Case Reports[pt] OR Comment[pt] OR Meta-Analysis[pt])	
**Embase**	248 articles
(‘conduction system pacing’ OR ‘biventricular pacing’ OR ‘left bundle branch pacing’ OR ‘his bundle pacing’) AND (RBBB or ‘right bundle branch block’ OR IVCD OR ‘intraventricular conduction delay’ OR ‘non-LBBB’ OR ‘non left bundle branch block’) AND (CRT-D OR ‘cardiac resynchronization therapy’/exp) NOT ([animals]/lim NOT [humans]/lim) NOT ([editorial]/lim OR [erratum]/lim OR [letter]/lim OR [note]/lim OR [review]/lim OR [short survey]/lim)	
**Scopus**	179 articles
(“conduction system pacing” OR “biventricular pacing” OR “left bundle branch pacing” OR “his bundle pacing”) AND (RBBB or “right bundle branch block” OR IVCD OR “intraventricular conduction delay” OR “non-LBBB” OR “non left bundle branch block”) AND (cardiac resynchronization therapy OR CRT-D) AND NOT (SRCTYPE (b) OR SRCTYPE (k) OR SRCTYPE (p) OR SRCTYPE (r) OR SRCTYPE (d) OR DOCTYPE (ab) OR DOCTYPE (bk) OR DOCTYPE (ch) OR DOCTYPE (bz) OR DOCTYPE (cp) OR DOCTYPE (cr) OR DOCTYPE (ed) OR DOCTYPE (er) OR DOCTYPE (le) OR DOCTYPE (no) OR DOCTYPE (pr) OR DOCTYPE (rp) OR DOCTYPE (re) OR DOCTYPE (sh))	

Supplementary Table S2. Risk of bias in included studies

	Selection	Comparability	Exposure	Total
Tan et al., 2023	***	**	***	8
Pujol-Lopez et al., 2022	**	**	***	7
Chen et al., 2023	***	**	***	8
Vijayaraman et al., 2022	***	*	***	7
